# A Muscarinic Antagonist Reduces Airway Inflammation and Bronchoconstriction Induced by Ambient Particulate Matter in a Mouse Model of Asthma

**DOI:** 10.3390/ijerph15061189

**Published:** 2018-06-06

**Authors:** Jun Kurai, Masanari Watanabe, Hiroyuki Sano, Kyoko Iwata, Degejirihu Hantan, Eiji Shimizu

**Affiliations:** 1 Department of Respiratory Medicine and Rheumatology, Faculty of Medicine, Tottori University, 36-1 Nishi-cho, Yonago, Tottori 683-8504, Japan; kurajun@med.tottori-u.ac.jp (J.K.); iwatak@mfc.or.jp (K.I.); degu.jirefu@technopro.com (D.H.); shimizu.eiji@hmw.gr.jp (E.S.); 2 Department of Respiratory Medicine and Allergology, Kinki University, 377-2 Ohnohigashi, Osakasayama, Osaka 589-0014, Japan; hsano@med.kindai.ac.jp; 3 Mio Fertility Clinic, Reproductive Centre, 2-2-1 Kuzumo-Minami, Yonago, Tottori 683-0008, Japan

**Keywords:** airway inflammation, asthma, muscarinic antagonists, ovalbumin mouse model, particulate matter

## Abstract

Ambient particulate matter (PM) can increase airway inflammation and induce bronchoconstriction in asthma. This study aimed to investigate the effect of tiotropium bromide, a long-acting muscarinic antagonist, on airway inflammation and bronchoconstriction induced by ambient PM in a mouse model of asthma. We compared the effect of tiotropium bromide to that of fluticasone propionate and formoterol fumarate. BALB/c mice were sensitized to ovalbumin (OVA) via the airways and then administered tiotropium bromide, fluticasone propionate, or formoterol fumarate. Mice were also sensitized to ambient PM via intranasal instillation. Differential leukocyte counts and the concentrations of interferon (IFN)-γ, interleukin (IL)-5, IL-6, IL-13, and keratinocyte-derived chemokine (KC/CXCL1) were measured in bronchoalveolar lavage fluid (BALF). Diacron-reactive oxygen metabolites (dROMs) were measured in the serum. Airway resistance and airway inflammation were evaluated in lung tissue 24 h after the OVA challenge. Ambient PM markedly increased neutrophilic airway inflammation in mice with OVA-induced asthma. Tiotropium bromide improved bronchoconstriction, and reduced neutrophil numbers, decreased the concentrations of IL-5, IL-6, IL-13, and KC/CXCL1 in BALF. However, tiotropium bromide did not decrease the levels of dROMs increased by ambient PM. Though eosinophilic airway inflammation was reduced with fluticasone propionate, neutrophilic airway inflammation was unaffected. Bronchoconstriction was improved with formoterol fumarate, but not with fluticasone propionate. In conclusion, tiotropium bromide reduced bronchoconstriction, subsequently leading to reduced neutrophilic airway inflammation induced by ambient PM.

## 1. Introduction

Numerous studies have elucidated the relationship between outdoor air pollution and the morbidity and mortality of cardiovascular and respiratory diseases [1,2]. It is also well established that short-term exposure to outdoor air pollution is associated with poor respiratory health, such as hospital admissions and emergency visits [3,4,5,6,7,8]. Similarly, outdoor air pollution is an important risk factor of exacerbation in asthma [9,10,11]. Thus, outdoor air pollution is a major environmental health problem affecting the majority of the population. This issue is aggravated by the inefficient combustion of fuels for transport and power generation.

Particulate matter (PM), also known as particle pollution, is a complex mixture of extremely small particles and liquid droplets, which are released into the air. Ambient PM is an important source of outdoor air pollution that has been associated with asthma exacerbations. The mechanisms of ambient PM-induced asthma exacerbations are increasingly being clarified. Several studies have clearly shown that ambient PM can increase airway inflammation in a mouse model of asthma by increasing the number of inflammatory cells in bronchoalveolar lavage fluid (BALF) and the concentrations of various chemokines and inflammatory mediators in lung tissue [12,13]. Neutrophils migrate to the lung during acute inflammation induced by exposure to ambient PM in humans [14]. Ambient PM also increases the concentration of IL-8 in BALF and IL-8 mRNA expression in bronchial biopsy tissue obtained from healthy subjects [15]. Similarly, our previous study revealed that ambient PM strongly induces neutrophilic airway inflammation through macrophage inflammatory protein (MIP)-2 (MIP-2/CXCL2), which is a murine homologue of IL-8, and IL-6 in a mouse model of asthma [12]. In another mouse model of asthma, exposure to ambient PM also induced neutrophilic airway inflammation accompanied by an increase in Th1 and Th17 cells [16]. Thus, neutrophilic airway inflammation may play an important role in the exacerbation of asthma induced by exposure to ambient PM.

The link between asthma and eosinophilic airway inflammation is well established [17]. Therefore, eosinophilic airway inflammation in asthma is strongly suppressed by corticosteroids. However, in some patients with asthma, neutrophilic airway inflammation dominates over eosinophilic airway inflammation [18]. Consequently, corticosteroids are only partially effective in these patients. In contrast, some researchers have reported that β_2_-agonists, which are also widely used in the treatment of asthma, can inhibit neutrophilic airway inflammation. For example, Bosmann et al. demonstrated that β_2_-agonists are able to reduce neutrophil recruitment to the lungs and inhibit the release of pro-inflammatory mediators [19]. Several studies have shown that muscarinic antagonists may attenuate eosinophilic airway inflammation and inhibit airway remodeling and hyperresponsiveness in vivo and in vitro [20,21,22]. However, it is largely unknown whether muscarinic antagonists have the potential to inhibit neutrophilic airway inflammation in asthma.

The present study investigated the effects of tiotropium bromide on airway inflammation and bronchoconstriction induced by ambient PM. For this purpose, we determined differential leukocyte counts and concentrations of IFN-γ, IL-5, IL-6, IL-13, and KC/CXCL1 in BALF in a mouse model of OVA-induced asthma. We also examined the histopathological findings and the mechanisms involved in the attenuation of tiotropium bromide airway inflammation. We hypothesized that tiotropium bromide would reduce bronchoconstriction and lead to reduced neutrophilic airway inflammation induced by ambient PM.

## 2. Materials and Methods

### 2.1. Animals

Specific pathogen-free 7-week-old male BALB/c mice were purchased from Charles River Laboratories Japan Inc. (Kanagawa, Japan) and acclimatized for 7 days before the start of the study. Animals were kept in a storage room at a constant temperature of 22 °C and illumination with 12-h light/dark cycles. Animals were fed standard animal chow daily and had *ad libitum* access to drinking water. The experimental protocols were approved by the Institutional Animal Care and Use Committee, Faculty of Medicine, Tottori University (protocol number 14-Y-46).

### 2.2. Preparation of Ambient PM

From 9 October 2015 to 30 October 2015, ambient PM was collected in Matsue city, the capital city of the Shimane Prefecture in southwest Japan. Total suspended particles were collected on a 20 × 25 cm quartz filter (2500QAT-UP; Tokyo Dylec, Tokyo, Japan) at a flow rate of 1000 L/min using a high-volume air sampler (HV-1000R; Shibata, Tokyo, Japan) for 23 h from 7 a.m. to 6 a.m. the following day. Before sampling, in order to remove endotoxins from filters, the filters were sterilized by dry heat at 240 °C for 30 min. After sampling, the 4-cm^2^ filter was detached and extracted with 4 mL of distilled deionized water and stored in a freezer at −20 °C to prevent growth of bacteria and fungi. For administration to mice, ambient PM was diluted with normal saline (NS) at an adequate concentration.

### 2.3. Experimental Protocol

Mice were sensitized to 20 μg of OVA (Sigma-Aldrich, St. Louis, MO, USA) emulsified in 2.25 mg of alum (Cosmo Bio Co., Ltd., Tokyo, Japan) by intraperitoneal injection or they received NS in a total volume of 100 μL on day 0 and day 14. On days 16 to 20, mice were also exposed to ambient PM (0.1 mg/25 μL of NS) or NS by intranasal instillation. Next, on days 21 to 26, mice were challenged with OVA (1% in NS) for 20 min via the airways by ultrasonic nebulization (Omron Healthcare Co., Ltd., Kyoto, Japan), followed by ambient PM exposure or NS exposure in the same manner on days 16 to 20. 

**Figure 1:**
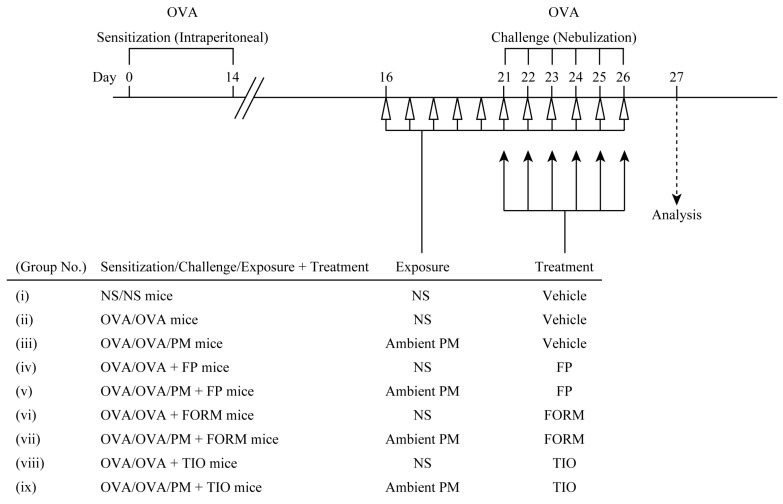
Experimental protocol of the mouse model of ovalbumin (OVA)-induced asthma used in the present study. For details, please see the text. FORM, formoterol fumarate; FP, fluticasone propionate; NS, normal saline; PM, particulate matter; TIO, tiotropium bromide.

To investigate the effect of drugs on airway inflammation and respiratory function, mice were treated with fluticasone propionate (Toronto Research Chemicals Inc., North York, ON, Canada), formoterol fumarate (Toronto Research Chemicals Inc.), or tiotropium bromide (Tokyo Chemical Industry Co., Ltd., Tokyo, Japan) on days 21 to 26. Mice in six groups, group (iv), (v), (vi), (vii), (viii), and (ix) as shown in Figure 1, received treatments with these drugs respectively as treatment groups. Other mice in three groups, group (i), (ii), and (iii), did not receive treatments as control groups. In the treatment groups, fluticasone propionate, a representative inhaled corticosteroid, was dissolved in 2% dimethyl sulfoxide in NS and administered intranasally at a volume of 50 μL (0.5 mg/mL) after OVA challenge exposure, followed by ambient PM exposure or NS exposure as previously described [23]. Formoterol fumarate, a representative long-acting β_2_-agonist, was dissolved in NS and administered intranasally at a volume of 50 μL (0.4 mg/kg) in the same order as furuticasone propionate [24].

Ohta et al. reported the anti-inflammatory effect of tiotropium bromide, a representative long-acting muscarinic antagonist, on airway inflammation in a mouse model of OVA-induced asthma at a concentration of 50 μg/mL via inhalation [22]. Therefore, tiotropium bromide was dissolved in NS and administered via the airways by ultrasonic nebulization at a concentration of 50 μg/mL for 3 min in the same order as furuticasone propionate and formoterol fumarate. Finally, the nine experimental groups were as follows: (i) NS/NS mice: sensitized to NS and challenged with NS; (ii) OVA/OVA mice: sensitized to OVA and challenged with OVA; (iii) OVA/OVA/PM mice: sensitized to OVA, challenged with OVA, and exposed to ambient PM; (iv) OVA/OVA+FP mice: sensitized to OVA, challenged with OVA, and treated with fluticasone propionate; (v) OVA/OVA/PM+FP mice: sensitized to OVA, challenged with OVA, exposed to ambient PM, and treated with fluticasone propionate; (vi) OVA/OVA+FORM mice: sensitized to OVA, challenged with OVA, and treated with formoterol fumarate; (vii) OVA/OVA/PM+FORM mice: sensitized to OVA, challenged with OVA, exposed to ambient PM, and treated with formoterol fumarate; (viii) OVA/OVA+TIO mice: sensitized to OVA, challenged with OVA, and treated with tiotropium bromide; and (ix) OVA/OVA/PM+TIO mice: sensitized to OVA, challenged with OVA, exposed to ambient PM, and treated with tiotropium bromide.Before sacrificing the animals on day 27, lung function was monitored in terms of specific airway resistance (sRaw) by plethysmography, followed by collection of BALF from the airways as well as whole blood and lung tissue.

### 2.4. BALF Procedure

After the mice were anesthetized with isoflurane, their tracheas were cannulated. BALF was obtained following instillation of 5 × 1.0 mL of NS into the lungs, along with gentle handling to maximize BALF recovery. BALF from each mouse was centrifuged at 300× *g* for 5 min at 4 °C. The cell pellets were used for cell counts and the supernatants were used for cytokine analysis. Total cells diluted in Turk’s fluid were counted using a hemocytometer. The differential leukocyte count was obtained by microscopic evaluation and quantitative analysis of methanol-fixed cytospin preparations stained with Diff Quick (Fisher Scientific, Pittsburgh, PA, USA).

### 2.5. Quantitative Determination of Cytokine Concentrations

The concentrations of IFN-γ, IL-5, IL-6, IL-13, and keratinocyte-derived chemokine (KC/CXCL1) in BALF were measured by using enzyme immunoassay (EIA) kits (R&D Systems Europe, Abingdon, UK). BALF was diluted 1/5 to determine the concentrations of IL-5, IL-6, IL-13, and KC/CXCL1. For IFN-γ, it was used undiluted. All EIA assays were performed according to the manufacturer’s instructions.

### 2.6. Histological Examination

Mice were euthanized by injection of pentobarbital. Lungs were inflation-fixed at 25 cm of water pressure with 10% formalin for 5 min and immersed in the same fixative. Tissues were fixed for 24 h at 4 °C and processed using standard methods for paraffin-embedded blocks. Fixed lung tissues were embedded in paraffin and each section was stained with hematoxylin and eosin (H&E).

### 2.7. Measurement of Airway Resistance

Airway resistance measurements were acquired at FinePointe™ Non-Invasive Airway Mechanics (NAM) sites (Buxco Electronics, Inc., Wilmington, NC, USA) using conscious mice [25]. Prior to measurement, mice were acclimated for 15 min to the chambers. The chambers were also calibrated each time before data collection. Briefly, the nasal chamber in combination with the thoracic chamber allowed computation of sRaw. FinePointe™ software computed sRaw values with all other ventilatory parameters such as frequency of breath, tidal volume, minute volume, inspiratory time, and expiratory time derived by the NAM analyzer.

### 2.8. Oxidative Stress Measurements

After the BALF procedure, whole blood was collected from the inferior vena cava. The blood samples were transferred into 1.5-mL tubes containing serum-separating medium (Bloodsepar; IBL, Gunma, Japan). After standing at room temperature for 30 min, the samples were centrifuged (3000 rpm, 10 min) and serum samples were collected. The samples were stored at −80 °C and transported on dry ice. All serum analyses were performed using a free radical analyzer system (FREE carpe diem, Wismerll Company Ltd., Tokyo, Japan) according to the manufacturer’s instructions. To analyze the serum levels of reactive oxygen metabolites, the levels of diacron reactive oxygen metabolites (dROMs) were measured. The results of dROM testing were expressed in arbitrary units (U. Carr), one unit corresponding to 0.8 mg/L of hydrogen peroxide as previously reported [26].

### 2.9. Statistical Analysis

Data are expressed as mean and standard deviation (SD). Comparisons between groups were conducted by one-way analysis of variance (ANOVA) with Turkey’s post-hoc tests. Calculations were performed with GraphPad Prism ver. 5.02 (GraphPad Software, San Diego, CA, USA). A *p*-value < 0.05 was considered to be statistically significant.

## 3. Results

### 3.1. Cell Counts in BALF

**Figure 2:**
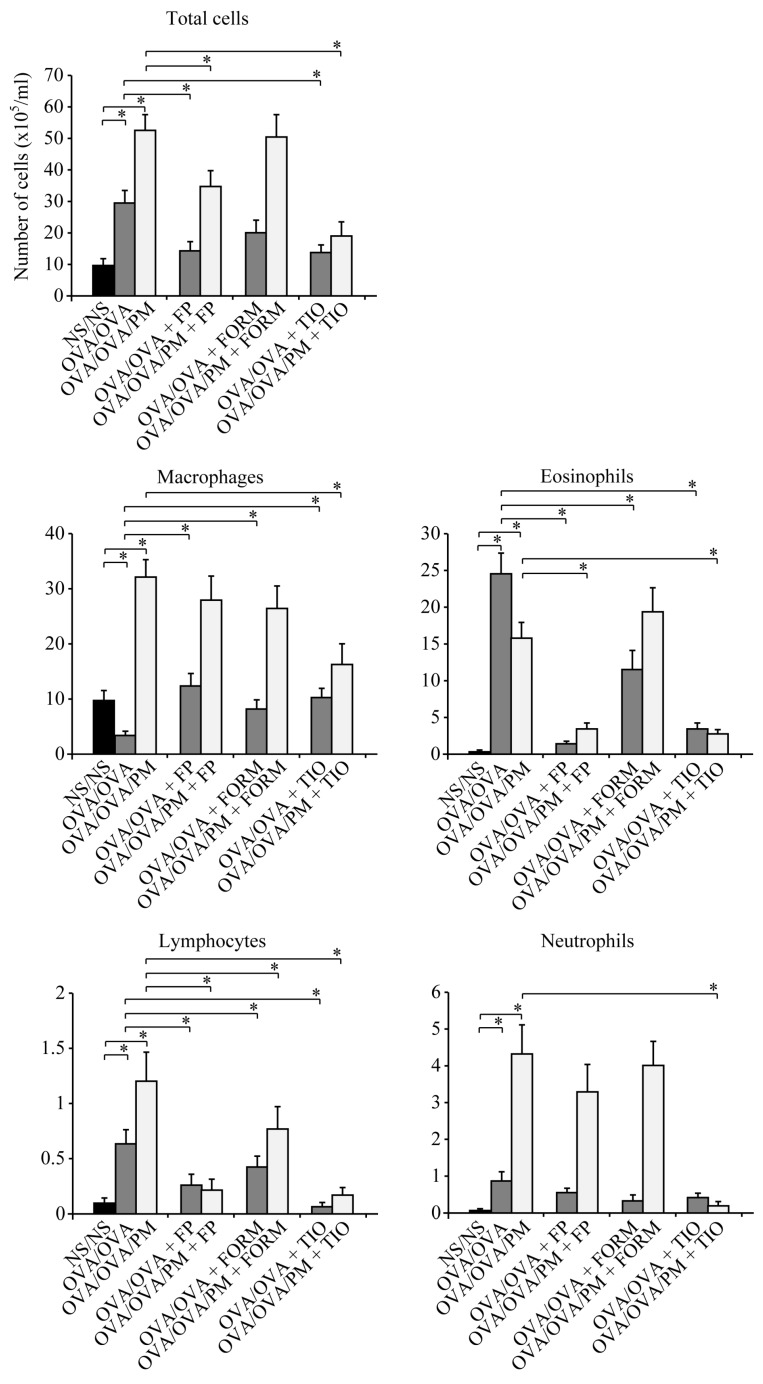
Total and differential leukocyte counts in bronchoalveolar lavage fluid (BALF). The cell counts in BALF were obtained 24 h after the final allergen challenge on day 26. The differential leukocyte counts included macrophages, lymphocytes, neutrophils, and eosinophils. The total cell count was significantly decreased by 35.1% in OVA/OVA/PM+FP mice and by 64.1% in OVA/OVA/PM+TIO mice, compared with OVA/OVA/PM mice. Data are expressed as the mean ± standard deviation, with eight mice per group. * *p* < 0.05.

OVA/OVA mice had a significantly higher BALF total cell count than control NS/NS mice (*p* < 0.05). OVA/OVA/PM mice had a 1.79-fold higher BALF total cell count than OVA/OVA mice (OVA/OVA/PM mice: 53.1 × 10^5^/mL; OVA/OVA mice: 29.6 × 10^5^/mL; *p* < 0.05). The increased cell count in OVA/OVA/PM mice compared with OVA/OVA mice was consistent for macrophages, lymphocytes, and neutrophils (Figure 2). In particular, macrophage and neutrophil cell counts were 9.29-fold (OVA/OVA/PM mice: 31.6 × 10^5^/mL; OVA/OVA mice: 3.43 × 10^5^/mL) and 4.95-fold (OVA/OVA/PM mice: 4.31 × 10^5^/mL; OVA/OVA mice: 0.87 × 10^5^/mL) higher in OVA/OVA/PM mice than in OVA/OVA mice (*p* < 0.05). Exposure to ambient PM increased the percentage of macrophages and neutrophils in total cells, which were 5.19-fold and 2.78-fold higher in OVA/OVA/PM mice than in OVA/OVA mice, respectively (Supplementary Figure S1).

OVA/OVA+FP mice and OVA/OVA+TIO mice had significantly lower BALF total cell counts than OVA/OVA mice (*p* < 0.05; Figure 2). Furthermore, both OVA/OVA/PM+FP mice and OVA/OVA/PM+TIO mice had significantly lower BALF total cell counts than OVA/OVA/PM mice (*p* < 0.05; Figure 2). The total cell count was significantly decreased by 35.1% in OVA/OVA/PM+FP mice and by 64.1% in OVA/OVA/PM+TIO mice (OVA/OVA/PM+TIO mice: 19.2 × 10^5^/mL; OVA/OVA/PM+FP mice: 34.7 × 10^5^/mL; OVA/OVA/PM mice: 53.1 × 10^5^/mL). The decreased cell counts were consistent for lymphocytes and eosinophils in OVA/OVA/PM+FP mice, lymphocytes in OVA/OVA/PM+FORM mice, and macrophages, lymphocytes, eosinophils, and neutrophils in OVA/OVA/PM+TIO mice, compared with OVA/OVA/PM mice (*p* < 0.05; Figure 2). Of note, a significantly lower neutrophil count (by 95.6%) was only observed in OVA/OVA/PM+TIO mice compared with OVA/OVA/PM mice (OVA/OVA/PM+TIO mice: 0.20 × 10^5^/mL; OVA/OVA/PM mice: 4.31 × 10^5^/mL; *p* < 0.05). Treatment with tiotropium bromide significantly reduced the percentage of neutrophils in total cell counts (*p* < 0.05; Supplementary Figure S1).

### 3.2. Cytokine Profile of BALF

**Figure 3:**
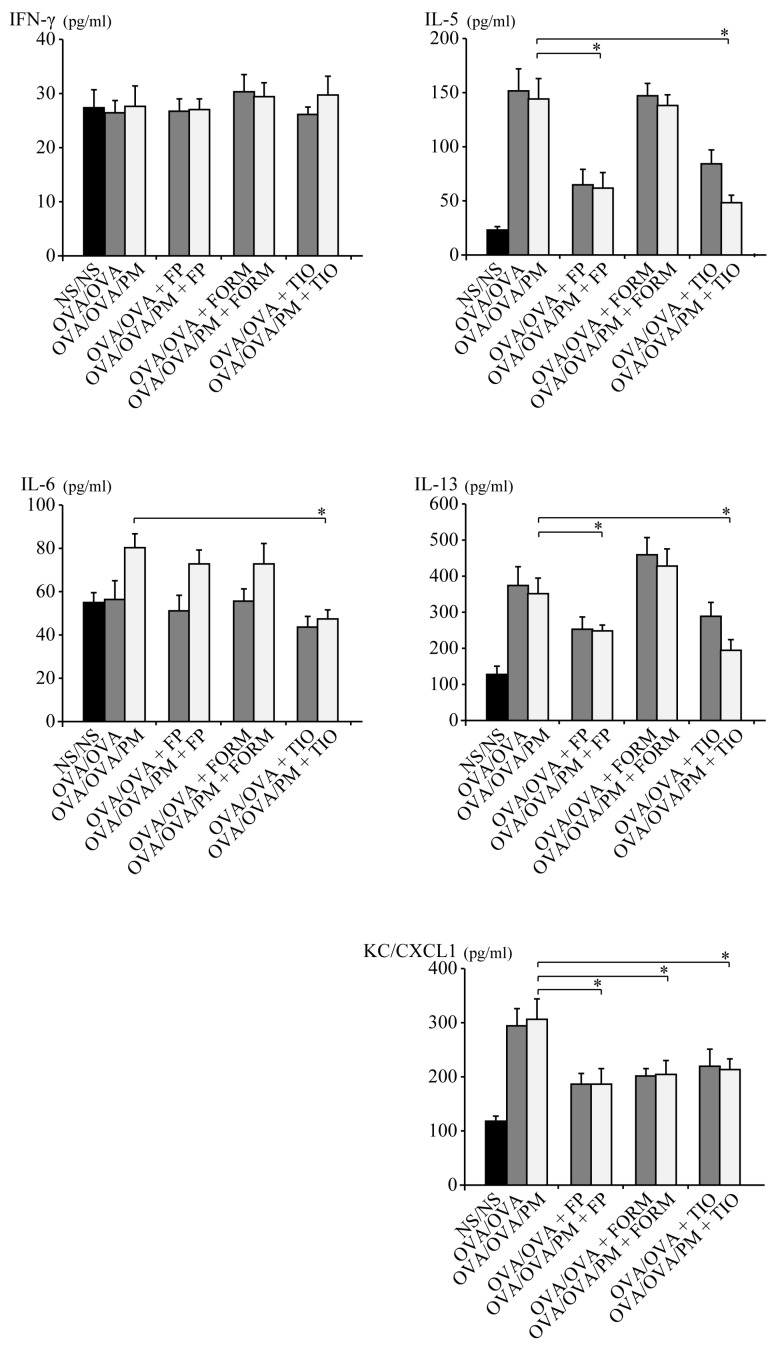
Cytokine concentrations in bronchoalveolar lavage fluid (BALF). BALF cytokine expression profiles were analyzed using enzyme immunoassays for IFN-γ, IL-5, IL-6, IL-13, and KC/CXCL1. Data for each group are expressed as the mean ± standard deviation, with six mice per group. * *p* < 0.05.

Cytokine concentrations in BALF were measured to investigate the mechanisms through which fluticasone propionate, formoterol fumarate, and tiotropium bromide attenuate the allergic airway response to ambient PM in OVA-induced asthma. In parallel with the inflammatory cell recruitment in BALF, ambient PM induced the production of several cytokines that are important in the development of asthma-related airway inflammation. The concentrations of IL-5 and IL-13 in OVA/OVA/PM+FP mice and OVA/OVA/PM+TIO mice were significantly lower than those in OVA/OVA/PM mice (*p* < 0.05; Figure 3). Additionally, the concentration of IL-6 was significantly more decreased in OVA/OVA/PM+TIO mice than in OVA/OVA/PM mice (*p* < 0.05; Figure 3). The concentration of IFN-γ was not affected by any treatment. The concentrations of IL-5, IL-6, IL-13, and IFN-γ were not affected in OVA/OVA/PM+FORM mice. The concentration of KC/CXCL1 was significantly decreased in OVA/OVA/PM+FP mice, OVA/OVA/PM+FORM mice, and OVA/OVA/PM+TIO mice compared to OVA/OVA/PM mice (*p* < 0.05; Figure 3).

### 3.3. Histopathological Changes in the Lung

**Figure 4:**
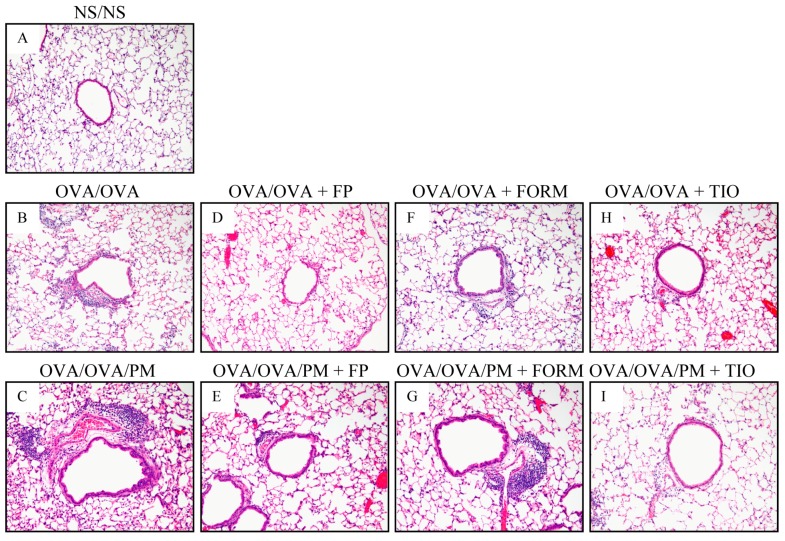
Effects of treatment of tiotropium bromide on histopathological changes in the lungs. Light photomicrographs of representative lung sections were stained using hematoxylin and eosin (magnification: ×200). Representative light photomicrographs of NS/NS mice (**A**); OVA/OVA mice (**B**); OVA/OVA/PM mice (**C**); OVA/OVA+FP mice (**D**); OVA/OVA/PM+FP mice (**E**); OVA/OVA+FORM mice (**F**); OVA/OVA/PM+FORM mice (**G**); OVA/OVA+TIO mice (**H**); and OVA/OVA/PM+TIO mice (**I**).

Lung specimens were stained with H&E to determine the histopathological effects of fluticasone propionate, formoterol fumarate, and tiotropium bromide on inflammatory cell infiltration. OVA/OVA mice had greater peribronchiolar and perivascular inflammatory cell infiltration compared with control NS/NS mice. Greater inflammatory cell infiltration was also apparent in OVA/OVA/PM mice compared with OVA/OVA mice (Figure 4). OVA/OVA/PM+FP mice and OVA/OVA/PM+TIO mice exhibited relatively weak inflammatory responses compared with OVA/OVA/PM mice. These histopathological findings were consistent with the BALF analysis, which revealed significant decreases in lymphocytes and eosinophils after treatment with fluticasone propionate, and in macrophages, lymphocytes, eosinophils, and neutrophils after treatment with tiotropium bromide. No anti-inflammatory responses were detected in OVA/OVA+FORM mice.

### 3.4. Measurement of Airway Resistance

**Figure 5:**
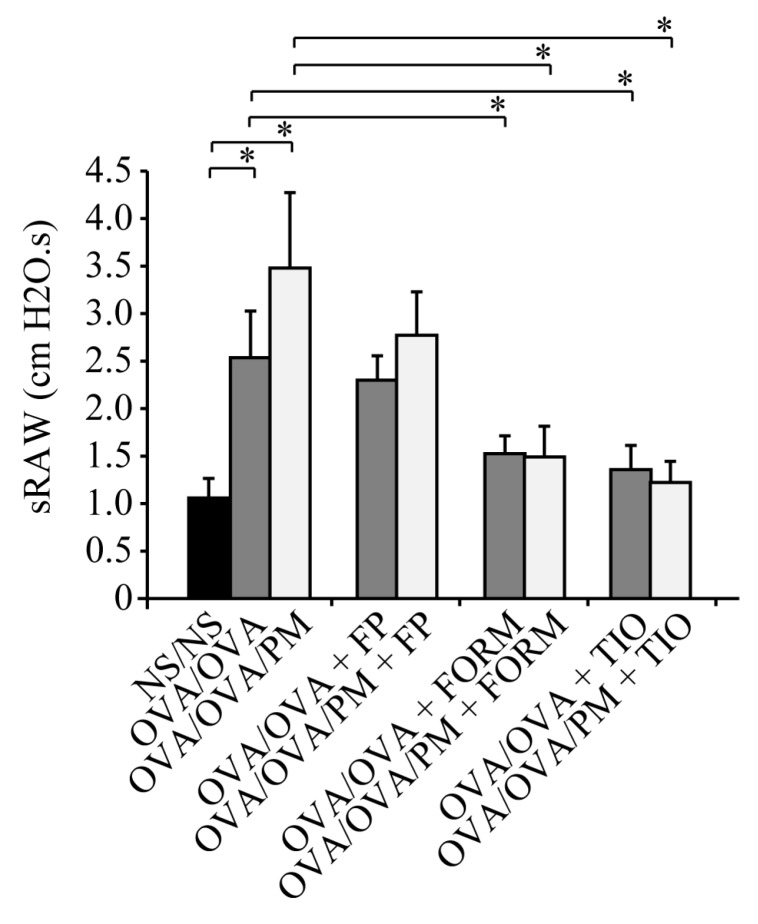
Effects of fluticasone propionate, formoterol fumarate, and tiotropium bromide on airway resistance. Airway resistance was evaluated by specific airway resistance (sRaw) values on day 27. Formoterol fumarate and tiotropium bromide decreased the sRaw values compared with fluticasone propionate. Data for each group are expressed as the mean ± standard deviation, with eight mice per group. * *p* < 0.05.

To assess the effects of fluticasone propionate, formoterol fumarate, and tiotropium bromide treatment on airway resistance induced by ambient PM in OVA-induced asthma, we measured sRaw on day 27. OVA/OVA mice showed a significant increase of the sRaw value compared with NS/NS mice. The sRaw value was significantly increased (1.37-fold) in OVA/OVA/PM mice compared with OVA/OVA mice. In contrast with OVA/OVA+FP mice and OVA/OVA/PM+FP mice, formoterol fumarate-treated mice challenged with OVA (OVA/OVA+FORM mice and OVA/OVA/PM+FORM mice) and tiotropium bromide-treated mice challenged with OVA (OVA/OVA+TIO mice and OVA/OVA/PM+TIO mice) had significantly lower sRaw values even in the presence or absence of ambient PM exposure (*p* < 0.05; Figure 5). The sRAW values of OVA/OVA/PM+TIO mice were decreased remarkably by 64.8% compared to OVA/OVA/PM mice, and the sRAW values of OVA/OVA/PM+FORM mice were decreased by 56.4% compared to OVA/OVA/PM mice (OVA/OVA/PM+TIO mice: 1.22 cm H_2_O.s; OVA/OVA/PM+FORM mice: 1.52 cm H_2_O.s; OVA/OVA/PM mice: 3.48 cm H_2_O.s; *p* < 0.05; Figure 5). While, the sRAW values of OVA/OVA/PM+FP were not decreased significantly compared to OVA/OVA/PM mice.

### 3.5. Measurement of dROMs in the Serum

**Figure 6:**
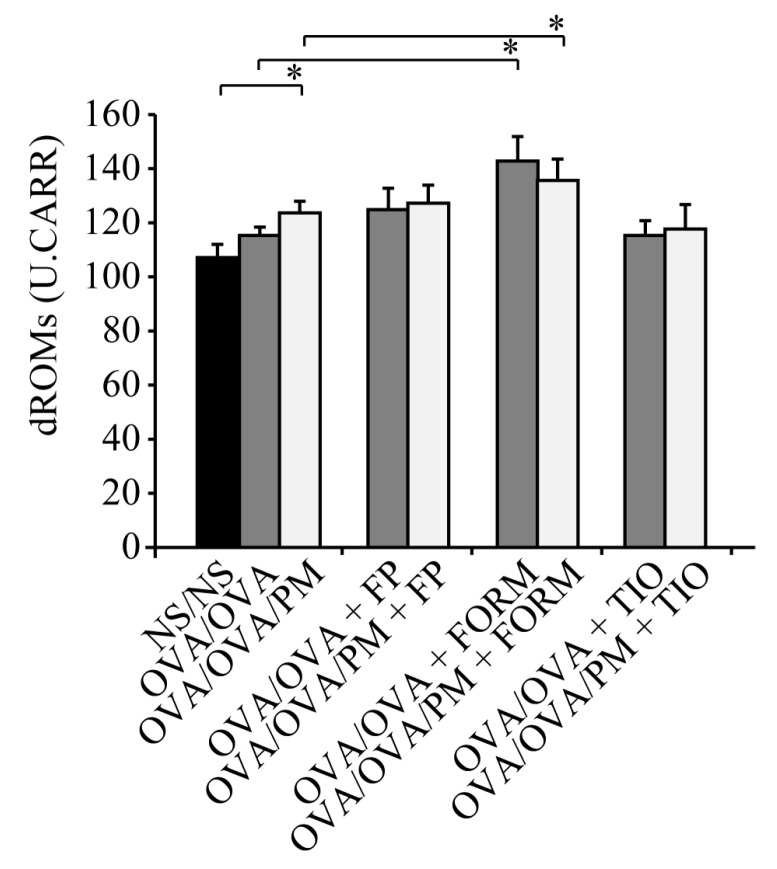
The levels of reactive oxygen metabolites after the administration of fluticasone propionate, formoterol fumarate, and tiotropium bromide. The levels of reactive oxygen metabolites (dROMs) in serum samples obtained on day 27. Fluticasone propionate and tiotropium bromide had no effect on dROM levels compared with the control group, but formoterol fumarate increased dROM levels. Data for each group are expressed as the mean ± standard deviation, with eight mice per group. * *p* < 0.05.

We measured dROMs in the serum to evaluate the implications of oxidative stress in airway inflammation increased by ambient PM exposure. Ambient PM exposure markedly increased the levels of dROMs in OVA/OVA/PM mice compared with control NS/NS mice (*p* < 0.05; Figure 6).

However, fluticasone propionate and tiotropium bromide had no effect on dROMs compared with OVA/OVA/PM mice. By contrast, both OVA/OVA+FORM mice and OVA/OVA/PM+FORM mice showed significantly increased dROM levels, raising the possibility that formoterol fumarate may increase rather than decrease oxidative stress (*p* < 0.05; Figure 6).

## 4. Discussion

In this study, ambient PM markedly increased neutrophilic airway inflammation in a mouse model of OVA-induced asthma. Together with the improvement of bronchoconstriction, tiotropium bromide attenuated neutrophilic airway inflammation augmented by ambient PM by decreasing the production of IL-5, IL-6, IL-13, and KC/CXCL1. In contrast, fluticasone propionate reduced eosinophilic airway inflammation, but not neutrophilic airway inflammation. Bronchoconstriction induced by ambient PM was improved by formoterol fumarate, but not by fluticasone propionate. These findings suggested that fluticasone propionate and tiotropium bromide may reduce airway inflammation augmented by ambient PM. However, only tiotropium bromide was able to inhibit neutrophilic airway inflammation whilst improving bronchoconstriction.

Tiotropium bromide acts as an antagonist of M3 muscarinic receptors on airway smooth muscles cells, thereby preventing binding of acetylcholine and subsequent bronchoconstriction [27]. Several recent studies have suggested that tiotropium bromide has anti-inflammatory effects in a mouse model of COPD [28,29]. For example, Wollin et al. revealed that tiotropium bromide significantly reduced the concentration of IL-6 and KC/CXCL1 and neutrophil cell counts in BALF of cigarette smoke-exposed mice [30]. However, only few published studies have focused on the beneficial effects of muscarinic antagonists using asthma mouse models [20,21,22]. To the best of our knowledge, none of the previously published studies has focused on neutrophilic airway inflammation. In the present study, we demonstrated that tiotropium bromide improved bronchoconstriction, and that it significantly reduced the concentrations of IL-5, IL-6, IL-13, and KC/CXCL1 in BALF, and subsequently led to reduced neutrophilic airway inflammation in a mouse model of OVA-induced asthma. In contrast, fluticasone propionate significantly reduced eosinophilic airway inflammation but had no effect on neutrophilic airway inflammation. Formoterol fumarate improved bronchoconstriction but was unable to reduce airway inflammation. These results suggest that, compared to fluticasone propionate and formoterol fumarate, tiotropium bromide inhibited neutrophilic airway inflammation augmented by ambient PM.

Exposure to air pollutants aggravates asthma symptoms and airway inflammation characterized by an increase in IL-6 and IL-8 [31,32,33]. These cytokines have important roles in neutrophilic inflammation in patients with asthma [34,35,36]. Our previous reports also showed that ambient PM increases neutrophilic airway inflammation and production of inflammatory IL-6 and MIP-2/CXCL2 in a mouse model of asthma [12]. In this study, the increased concentration of KC/CXCL1 by ambient PM in BALF was significantly decreased by tiotropium bromide, fluticasone propionate, and formoterol fumarate. However, the increased concentration of IL-6 was significantly decreased by tiotropium bromide, but not by fluticasone propionate or formoterol fumarate. Thus, tiotropium bromide was able to inhibit the increase of both IL-6 and KC/CXCL1 by ambient PM. The inhibition of both IL-6 and KC/CXCL1 may be important in decreasing neutrophilic airway inflammation augmented by ambient PM.

Inflammation and oxidative stress are closely linked to responses to ambient PM and are thought to be responsible for the majority of its adverse health effects [37,38]. An epidemiological study has shown that exposure of children to air pollution is associated with an increase in the oxidative stress markers, thiobarbituric acid-reactive substances [31]. In recent years, several publications have linked ambient PM exposure to the generation of reactive oxygen species (ROS) in pulmonary epithelial cells [39,40,41]. Vacca et al. reported that a muscarinic antagonist could reduce ROS release from human alveolar macrophages and reduce airway inflammation in vitro [42]. Therefore, we hypothesized that one mechanism underlying the effects of ambient PM on airway epithelial cells could involve the generation of ROS. In our study, ambient PM exposure increased ROS production, as indicated by the levels of dROMs. However, tiotropium bromide did not suppress ROS production. Therefore, our hypothesis was not supported. Further studies are needed to provide the mechanism whereby tiotropium bromide contributes to inhibition of neutrophilic airway inflammation.

It has been suggested that β_2_-agonists have anti-inflammatory properties against LPS-induced neutrophilic airway inflammation in addition to their conventional action on respiratory function improvement [19,43]. In contrast to previous reports, in the present study, we could not demonstrate the anti-inflammatory effects of formoterol fumarate against neutrophilic airway inflammation, yet bronchoconstriction was improved. Ambient PM is not a simple substance, such as LPS, but rather a complex mixture containing geological minerals, biological materials, and fossil fuel combustion products. Therefore, formoterol fumarate may not have beneficial effects on neutrophilic airway inflammation augmented by ambient PM.

The number of eosinophils in the BALF of OVA/OVA/PM mice was lower than in that of OVA/OVA mice. The mechanism underlying this process is not well understood. In contrast to eosinophils, the number of macrophages in OVA/OVA/PM mice significantly increased compared to OVA/OVA mice. Macrophages are phagocytes that play a critical role in host defense against foreign substances such as PM [44]. Therefore, exogenous material can increase the number of macrophages, for example in smokers or long-term city dwellers. The reason for the lower number of eosinophils in OVA/OVA/PM than in OVA/OVA mice may be that the robust induction of macrophages by PM exposure prevents the increase of eosinophils in BALF.

This study has several limitations. First, ambient PM is a complex mixture of various substances, and the effects of seasonal variation and regional heterogeneity on adverse health effects have been described [45,46,47,48]. Therefore, we should confirm the anti-inflammatory effects of tiotropium bromide by collecting ambient PM on different days and regions. Second, previous studies have shown significant increases in AHR to bronchoconstriction agents in a mouse model of OVA-induced asthma [49,50,51]. However, we did not evaluate AHR in the present study, as we were unable to collect a sufficient amount of ambient PM. Third, we could not measure the levels of dROMs in BALF to evaluate the implications of oxidative stress in airway inflammation increased by ambient PM exposure because we did not have a sufficient amount of BALF samples.

## 5. Conclusions

Tiotropium bromide, but not fluticasone propionate and formoterol fumarate, reduced bronchoconstriction and subsequently led to reduced neutrophilic airway inflammation augmented by ambient PM. Our data support the view that the positive effect of tiotropium bromide on asthma-related airway inflammation augmented by ambient PM can be partly attributed to its anti-inflammatory activity.

## Supplementary Materials

The following are available online at http://www.mdpi.com/1660-4601/15/6/1189/s1. Figure S1: Percentage of macrophages, lymphocytes, neutrophils, and eosinophils in total cells in bronchoalveolar lavage fluid (BALF).

## Abbreviations

The following abbreviations are used in this manuscript:

PM	particulate matter
OVA	ovalbumin
IFN	interferon
IL	interleukin
KC/CXCL1	keratinocyte-derived chemokine
BALF	bronchoalveolar lavage fluid
dROMs	diacron reactive oxygen metabolites
MIP-2/CXCL2	macrophage inflammatory protein
NS	normal saline
FORM	formoterol fumarate
FP	fluticasone propionate
TIO	tiotropium bromide
sRAW	specific airway resistance
EIA	enzyme immunoassay
H&E	hematoxylin and eosin
SD	standard deviation
ROS	reactive oxygen species

## References

[1] Ware J.H., Thibodeau L.A., Speizer F.E., Colome S., Ferris B.G. (1981). Assessment of the health effects of atmospheric sulfur oxides and particulate matter: Evidence from observational studies. Environ. Health Perspect..

[2] Dockery D.W., Pope C.A., Xu X., Spengler J.D., Ware J.H., Fay M.E., Ferris B.G., Speizer F.E. (1993). An association between air pollution and mortality in six U.S. Cities. N. Engl. J. Med..

[3] Yamazaki S., Shima M., Yoda Y., Oka K., Kurosaka F., Shimizu S., Takahashi H., Nakatani Y., Nishikawa J., Fujiwara K. (2014). Association between PM2.5 and primary care visits due to asthma attack in Japan: Relation to Beijing’s air pollution episode in January 2013. Environ. Health Prev. Med..

[4] Watanabe M., Yamasaki A., Burioka N., Kurai J., Yoneda K., Yoshida A., Igishi T., Fukuoka Y., Nakamoto M., Takeuchi H. (2011). Correlation between asian dust storms and worsening asthma in western Japan. Allergol. Int..

[5] Watanabe M., Kurai J., Igishi T., Yamasaki A., Burioka N., Takeuchi H., Sako T., Touge H., Nakamoto M., Hasegawa Y. (2012). Influence of asian desert dust on lower respiratory tract symptoms in patients with asthma over 4 years. Yonago Acta Med..

[6] Pope C.A., Burnett R.T., Thun M.J., Calle E.E., Krewski D., Ito K., Thurston G.D. (2002). Lung cancer, cardiopulmonary mortality, and long-term exposure to fine particulate air pollution. JAMA.

[7] Dominici F., Peng R.D., Bell M.L., Pham L., McDermott A., Zeger S.L., Samet J.M. (2006). Fine particulate air pollution and hospital admission for cardiovascular and respiratory diseases. JAMA.

[8] Mustafic H., Jabre P., Caussin C., Murad M.H., Escolano S., Tafflet M., Perier M.C., Marijon E., Vernerey D., Empana J.P. (2012). Main air pollutants and myocardial infarction: A systematic review and meta-analysis. JAMA.

[9] Li S., Williams G., Jalaludin B., Baker P. (2012). Panel studies of air pollution on children’s lung function and respiratory symptoms: A literature review. J. Asthma Off. J. Assoc. Care Asthma.

[10] McCreanor J., Cullinan P., Nieuwenhuijsen M.J., Stewart-Evans J., Malliarou E., Jarup L., Harrington R., Svartengren M., Han I.K., Ohman-Strickland P. (2007). Respiratory effects of exposure to diesel traffic in persons with asthma. N. Engl. J. Med..

[11] Zheng X.Y., Ding H., Jiang L.N., Chen S.W., Zheng J.P., Qiu M., Zhou Y.X., Chen Q., Guan W.J. (2015). Association between air pollutants and asthma emergency room visits and hospital admissions in time series studies: A systematic review and meta-analysis. PLoS ONE.

[12] Kurai J., Watanabe M., Tomita K., Yamasaki H.S., Shimizu E. (2014). Influence of asian dust particles on immune adjuvant effects and airway inflammation in asthma model mice. PLoS ONE.

[13] Ichinose T., Yoshida S., Sadakane K., Takano H., Yanagisawa R., Inoue K., Nishikawa M., Mori I., Kawazato H., Yasuda A. (2008). Effects of Asian sand dust, arizona sand dust, amorphous silica and aluminum oxide on allergic inflammation in the murine lung. Inhal. Toxicol..

[14] Sierra-Vargas M.P., Guzman-Grenfell A.M., Blanco-Jimenez S., Sepulveda-Sanchez J.D., Bernabe-Cabanillas R.M., Cardenas-Gonzalez B., Ceballos G., Hicks J.J. (2009). Airborne particulate matter PM2.5 from mexico city affects the generation of reactive oxygen species by blood neutrophils from asthmatics: An in vitro approach. J. Occup. Med. Toxicol..

[15] Frampton M.W., Utell M.J., Zareba W., Oberdorster G., Cox C., Huang L.S., Morrow P.E., Lee F.E., Chalupa D., Frasier L.M. (2004). Effects of exposure to ultrafine carbon particles in healthy subjects and subjects with asthma. Res. Rep. Health Eff. Inst..

[16] Kim Y.S., Choi E.J., Lee W.H., Choi S.J., Roh T.Y., Park J., Jee Y.K., Zhu Z., Koh Y.Y., Gho Y.S. (2013). Extracellular vesicles, especially derived from gram-negative bacteria, in indoor dust induce neutrophilic pulmonary inflammation associated with both th1 and th17 cell responses. Clin. Exp. Allergy.

[17] Kay A.B. (2005). The role of eosinophils in the pathogenesis of asthma. Trends Mol. Med..

[18] Drews A.C., Pizzichini M.M., Pizzichini E., Pereira M.U., Pitrez P.M., Jones M.H., Sly P.D., Stein R.T. (2009). Neutrophilic airway inflammation is a main feature of induced sputum in nonatopic asthmatic children. Allergy.

[19] Bosmann M., Grailer J.J., Zhu K., Matthay M.A., Sarma J.V., Zetoune F.S., Ward P.A. (2012). Anti-inflammatory effects of beta2 adrenergic receptor agonists in experimental acute lung injury. FASEB J. Off. Publ. Fed. Am. Soc. Exp. Biol..

[20] Bos I.S., Gosens R., Zuidhof A.B., Schaafsma D., Halayko A.J., Meurs H., Zaagsma J. (2007). Inhibition of allergen-induced airway remodelling by tiotropium and budesonide: A comparison. Eur. Respir. J..

[21] Gosens R., Bos I.S., Zaagsma J., Meurs H. (2005). Protective effects of tiotropium bromide in the progression of airway smooth muscle remodeling. Am. J. Respir. Crit. Care Med..

[22] Ohta S., Oda N., Yokoe T., Tanaka A., Yamamoto Y., Watanabe Y., Minoguchi K., Ohnishi T., Hirose T., Nagase H. (2010). Effect of tiotropium bromide on airway inflammation and remodelling in a mouse model of asthma. Clin. Exp. Allergy.

[23] Kimura G., Ueda K., Eto S., Watanabe Y., Masuko T., Kusama T., Barnes P.J., Ito K., Kizawa Y. (2013). Toll-like receptor 3 stimulation causes corticosteroid-refractory airway neutrophilia and hyperresponsiveness in mice. Chest.

[24] Hatchwell L., Girkin J., Dun M.D., Morten M., Verrills N., Toop H.D., Morris J.C., Johnston S.L., Foster P.S., Collison A. (2014). Salmeterol attenuates chemotactic responses in rhinovirus-induced exacerbation of allergic airways disease by modulating protein phosphatase 2a. J. Allergy Clin. Immunol..

[25] Glaab T., Taube C., Braun A., Mitzner W. (2007). Invasive and noninvasive methods for studying pulmonary function in mice. Respir. Res..

[26] Cornelli U., Terranova R., Luca S., Cornelli M., Alberti A. (2001). Bioavailability and antioxidant activity of some food supplements in men and women using the d-roms test as a marker of oxidative stress. J. Nutr..

[27] Disse B., Speck G.A., Rominger K.L., Witek T.J., Hammer R. (1999). Tiotropium (spiriva): Mechanistical considerations and clinical profile in obstructive lung disease. Life Sci..

[28] Cui Y., Devillier P., Kuang X., Wang H., Zhu L., Xu Z., Xia Z., Zemoura L., Advenier C., Chen H. (2010). Tiotropium reduction of lung inflammation in a model of chronic gastro-oesophageal reflux. Eur. Respir. J..

[29] Arai N., Kondo M., Izumo T., Tamaoki J., Nagai A. (2010). Inhibition of neutrophil elastase-induced goblet cell metaplasia by tiotropium in mice. Eur. Respir. J..

[30] Wollin L., Pieper M.P. (2010). Tiotropium bromide exerts anti-inflammatory activity in a cigarette smoke mouse model of copd. Pulm. Pharmacol. Ther..

[31] Liu L., Poon R., Chen L., Frescura A.M., Montuschi P., Ciabattoni G., Wheeler A., Dales R. (2009). Acute effects of air pollution on pulmonary function, airway inflammation, and oxidative stress in asthmatic children. Environ. Health Perspect..

[32] Bellido-Casado J., Plaza V., Perpina M., Picado C., Bardagi S., Martinez-Bru C., Torrejon M. (2010). (inflammatory response of rapid onset asthma exacerbation). Arch. Bronconeumol..

[33] Wittkopp S., Staimer N., Tjoa T., Gillen D., Daher N., Shafer M., Schauer J.J., Sioutas C., Delfino R.J. (2013). Mitochondrial genetic background modifies the relationship between traffic-related air pollution exposure and systemic biomarkers of inflammation. PLoS ONE.

[34] Wood L.G., Baines K.J., Fu J., Scott H.A., Gibson P.G. (2012). The neutrophilic inflammatory phenotype is associated with systemic inflammation in asthma. Chest.

[35] Pepe C., Foley S., Shannon J., Lemiere C., Olivenstein R., Ernst P., Ludwig M.S., Martin J.G., Hamid Q. (2005). Differences in airway remodeling between subjects with severe and moderate asthma. J. Allergy Clin. Immunol..

[36] Shannon J., Ernst P., Yamauchi Y., Olivenstein R., Lemiere C., Foley S., Cicora L., Ludwig M., Hamid Q., Martin J.G. (2008). Differences in airway cytokine profile in severe asthma compared to moderate asthma. Chest.

[37] Todokoro M., Mochizuki H., Tokuyama K., Utsugi M., Dobashi K., Mori M., Morikawa A. (2004). Effect of ozone exposure on intracellular glutathione redox state in cultured human airway epithelial cells. Inflammation.

[38] Peden D.B., Boehlecke B., Horstman D., Devlin R. (1997). Prolonged acute exposure to 0.16 ppm ozone induces eosinophilic airway inflammation in asthmatic subjects with allergies. J. Allergy Clin. Immunol..

[39] Zou Y., Jin C., Su Y., Li J., Zhu B. (2016). Water soluble and insoluble components of urban PM2.5 and their cytotoxic effects on epithelial cells (a549) in vitro. Environ. Pollut..

[40] Lee K.W., Nam M.H., Lee H.R., Hong C.O., Lee K.W. (2017). Protective effects of chebulic acid on alveolar epithelial damage induced by urban particulate matter. BMC Complement. Altern. Med..

[41] Zhou W., Tian D., He J., Wang Y., Zhang L., Cui L., Jia L., Zhang L., Li L., Shu Y. (2016). Repeated PM2.5 exposure inhibits beas-2b cell p53 expression through ros-akt-dnmt3b pathway-mediated promoter hypermethylation. Oncotarget.

[42] Vacca G., Randerath W.J., Gillissen A. (2011). Inhibition of granulocyte migration by tiotropium bromide. Respir. Res..

[43] Maris N.A., de Vos A.F., Dessing M.C., Spek C.A., Lutter R., Jansen H.M., van der Zee J.S., Bresser P., van der Poll T. (2005). Antiinflammatory effects of salmeterol after inhalation of lipopolysaccharide by healthy volunteers. Am. J. Respir. Crit. Care Med..

[44] Lambrecht B.N. (2006). Alveolar macrophage in the driver’s seat. Immunity.

[45] Kurai J., Watanabe M., Sano H., Hantan D., Shimizu E. (2016). The effect of seasonal variations in airborne particulate matter on asthma-related airway inflammation in mice. Int. J. Environ. Res. Public Health.

[46] Bell M.L., Ebisu K., Peng R.D., Walker J., Samet J.M., Zeger S.L., Dominici F. (2008). Seasonal and regional short-term effects of fine particles on hospital admissions in 202 US counties, 1999–2005. Am. J. Epidemiol..

[47] Dominici F., McDermott A., Zeger S.L., Samet J.M. (2003). National maps of the effects of particulate matter on mortality: Exploring geographical variation. Environ. Health Perspect..

[48] Franklin M., Zeka A., Schwartz J. (2007). Association between PM2.5 and all-cause and specific-cause mortality in 27 US communities. J. Expo. Sci. Environ. Epidemiol..

[49] Wang X., Hui Y., Zhao L., Hao Y., Guo H., Ren F. (2017). Oral administration of lactobacillus paracasei l9 attenuates PM2.5-induced enhancement of airway hyperresponsiveness and allergic airway response in murine model of asthma. PLoS ONE.

[50] Penton P.C., Wang X., Amatullah H., Cooper J., Godri K., North M.L., Khanna N., Scott J.A., Chow C.W. (2013). Spleen tyrosine kinase inhibition attenuates airway hyperresponsiveness and pollution-induced enhanced airway response in a chronic mouse model of asthma. J. Allergy Clin. Immunol..

[51] Wang T., Moreno-Vinasco L., Huang Y., Lang G.D., Linares J.D., Goonewardena S.N., Grabavoy A., Samet J.M., Geyh A.S., Breysse P.N. (2008). Murine lung responses to ambient particulate matter: Genomic analysis and influence on airway hyperresponsiveness. Environ. Health Perspect..

