# Supplementary material for: A Muscarinic Antagonist Reduces Airway Inflammation and Bronchoconstriction Induced by Ambient Particulate Matter in a Mouse Model of Asthma
Source: Int J Environ Res Public Health. 2018 Jun 6;15(6):1189. doi: 10.3390/ijerph15061189 (PMC6025324; doi:10.3390/ijerph15061189)
Supplement: Supplementary file 1 [file ijerph-15-01189-s001.pdf]

Supplementary Material

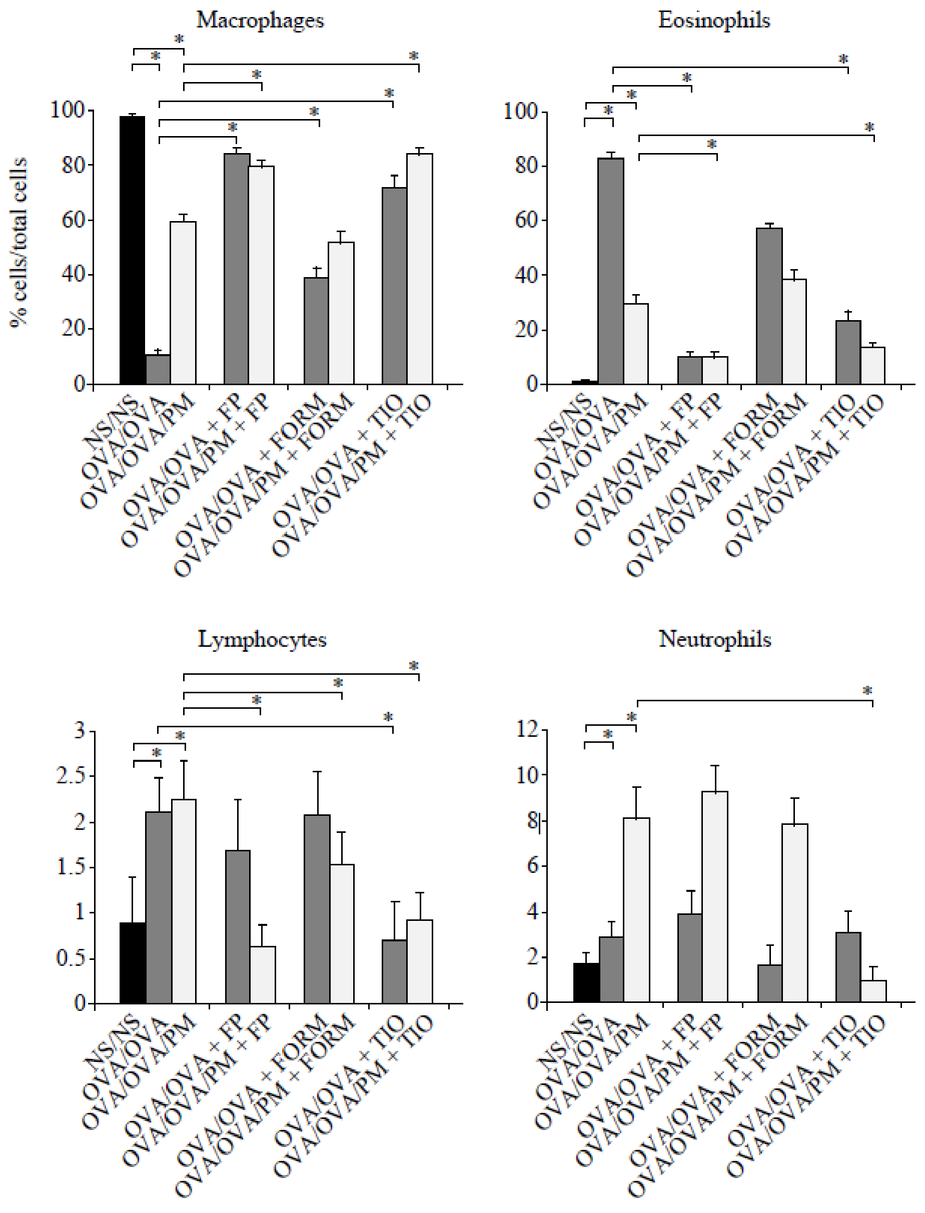

Figure S1: Percentage of macrophages, lymphocytes, neutrophils, and eosinophils in total cells in bronchoalveolar lavage fluid (BALF)
